# Supplementary material for: Effect of COVID-19 pandemic on internet gaming disorder among general population: A systematic review and meta-analysis
Source: PLOS Glob Public Health. 2023 Apr 7;3(4):e0001783. doi: 10.1371/journal.pgph.0001783 (PMC10081738; doi:10.1371/journal.pgph.0001783)
Supplement: S2 Table — (DOCX) [file pgph.0001783.s002.docx]

**S2 Table. Search strategies in different search engines and registry database.**

| Search engines, Registry database | Search terms |
| --- | --- |
| PubMed | ((("online gam*"[Title/Abstract] OR "internet gam*"[Title/Abstract] OR "computer gam*"[Title/Abstract] OR "video gam*"[Title/Abstract] OR "gaming"[Title/Abstract] OR "online role-playing game"[Title/Abstract] OR "gaming behavior"[Title/Abstract] OR "digital gam*"[Title/Abstract])) AND (("abuse"[Title/Abstract] OR "addiction"[Title/Abstract] OR "compulsive"[Title/Abstract] OR "dependenc*"[Title/Abstract] OR "disorder*"[Title/Abstract] OR "effects"[Title/Abstract] OR "excessive"[Title/Abstract] OR "habits"[Title/Abstract] OR "misuse"[Title/Abstract] OR "pathological"[Title/Abstract] OR "problem"[Title/Abstract] OR "problematic"[Title/Abstract] OR "overuse"[Title/Abstract] OR "Internet Addiction Disorder"[MeSH Terms]))) AND (("COVID-19"[MeSH Terms] OR "COVID-19"[Title/Abstract] OR "covid 19 pandemic*"[Title/Abstract]))  Filters used: English, 2020-2022 |
| EMBASE | ('coronavirus disease 2019'/exp OR 'coronavirus disease 2019':ab,ti OR 'covid 19':ab,ti) AND ('online gaming':ab,ti OR 'internet gaming':ab,ti OR 'computer gaming':ab,ti OR 'video gaming':ab,ti OR 'gaming':ab,ti OR 'online-role playing gaming':ab,ti OR 'gaming behavior':ab,ti OR 'digital gaming':ab,ti) AND (abuse:ab,ti OR addiction:ab,ti OR compulsive:ab,ti OR dependence:ab,ti OR disorder:ab,ti OR effects:ab,ti OR excessive:ab,ti OR habit:ab,ti OR misuse:ab,ti OR overuse:ab,ti OR problem:ab,ti) AND [humans]/lim AND [english]/lim AND [embase]/lim AND [2020-2022]/py  Filters used: English, 2020-2022 |
| Scopus | ( TITLE-ABS-KEY ( "online gam*" OR "internet gam*" OR "computer gam*" OR "video gam*" OR "gaming" OR "online role-playing game" OR "gaming behavior" OR "digital gam*" ) AND TITLE-ABS-KEY ( abuse OR addiciton OR compulsive OR dependenc* OR disorder* OR effect* OR excessive OR habit* OR misuse OR pathological OR problem* OR problematic OR overuse ) AND TITLE-ABS-KEY ( "COVID-19" OR "COVID 19" OR "COVID 19 pandemic" ) ) AND ( LIMIT-TO ( PUBYEAR , 2022 ) OR LIMIT-TO ( PUBYEAR , 2021 ) OR LIMIT-TO ( PUBYEAR , 2020 ) ) AND ( LIMIT-TO ( LANGUAGE , "English" ) ) AND ( LIMIT-TO ( EXACTKEYWORD , "Human" ) OR LIMIT-TO ( EXACTKEYWORD , "Humans" ) ) Filters used: Human, English, 2020-2022 |
| CINAHL | ( TI "online gam*" OR AB "online gam*" OR TI "internet gam*" OR AB "internet gam*" OR TI "computer gam*" OR AB "computer gam*" OR TI "video gam*" OR AB "video gam*" OR TI "online-role playing game" OR AB "online-role playing game" OR TI "gaming behavio?r" OR AB "gaming behavio?r" OR TI "digital gam*" OR AB "digital gam*" ) AND ( TI abuse OR AB abuse OR TI addiction OR AB addiction OR TI compulsive OR AB compulsive OR TI dependenc* OR AB dependenc* OR TI disorder* OR AB disorder* OR TI effect* OR AB effect* OR TI excessive OR AB excessive OR TI habit* OR AB habit* OR TI misuse* OR AB misuse* OR TI pathological OR AB pathological OR TI problem* OR AB problem* OR TI overuse OR AB overuse ) AND ( (MH "COVID-19") OR TI "COVID-19" OR AB "COVID-19" OR TI "COVID 19" OR AB "COVID 19" OR TI "COVID 19 pandemic" OR AB "COVID 19 pandemic" )  Filters used: English and Pub Date: 2020-2022 |
| PsycNet | ((title: ("online gam*")) OR (abstract: ("online gam*")) OR (title: ("internet gam*")) OR (abstract: ("internet gam*")) OR (title: ("computer gam*")) OR (abstract: ("computer gam*")) OR (title: ("video gam*")) OR (abstract: ("video gam*")) OR (title: ("online role-playing game")) OR (abstract: ("online role-playing game")) OR (title: ("gaming behaviour")) OR (abstract: ("gaming behaviour")) OR (title: ("digital gam*")) AND (abstract: ("digital gam*"))) AND (((title: (abuse)) OR (abstract: (abuse)) OR (title: (addiciton)) OR (abstract: (addiciton)) OR (title: (compulsive)) OR (abstract: (compulsive)) OR (title: (dependenc*)) OR (abstract: (dependenc*)) OR (title: (disorder*)) OR (abstract: (disorder*)) OR (title: (effect*)) OR (abstract: (effect*)) OR (title: (excessive)) OR (abstract: (excessive)) OR (title: (habit*)) OR (abstract: (habit*)) OR (title: (misuse)) OR (abstract: (misuse)) OR (title: (pathological)) OR (abstract: (pathological)) OR (title: (problem*)) OR (abstract: (problem*)) OR (title: (problematic)) OR (abstract: (problematic)) OR (title: (overuse)) OR (abstract: (overuse))) AND (HasFullText: true)) AND ((title: ("COVID-19")) OR (abstract: ("COVID-19")) OR (title: ("COVID 19")) OR (abstract: ("COVID 19")) OR (title: ("COVID 19 pandemic")) OR (abstract: ("COVID 19 pandemic")))  Filters used: English and Pub Date: 2020-2022 |

Table 1: Search strategies in different search engines and registry database

| Search engines, Registry database | Search terms |
| --- | --- |
| PubMed | ((("online gam*"[Title/Abstract] OR "internet gam*"[Title/Abstract] OR "computer gam*"[Title/Abstract] OR "video gam*"[Title/Abstract] OR "gaming"[Title/Abstract] OR "online role-playing game"[Title/Abstract] OR "gaming behavior"[Title/Abstract] OR "digital gam*"[Title/Abstract])) AND (("abuse"[Title/Abstract] OR "addiction"[Title/Abstract] OR "compulsive"[Title/Abstract] OR "dependenc*"[Title/Abstract] OR "disorder*"[Title/Abstract] OR "effects"[Title/Abstract] OR "excessive"[Title/Abstract] OR "habits"[Title/Abstract] OR "misuse"[Title/Abstract] OR "pathological"[Title/Abstract] OR "problem"[Title/Abstract] OR "problematic"[Title/Abstract] OR "overuse"[Title/Abstract] OR "Internet Addiction Disorder"[MeSH Terms]))) AND (("COVID-19"[MeSH Terms] OR "COVID-19"[Title/Abstract] OR "covid 19 pandemic*"[Title/Abstract]))  Filters used: English, 2020-2022 |
| EMBASE | ('coronavirus disease 2019'/exp OR 'coronavirus disease 2019':ab,ti OR 'covid 19':ab,ti) AND ('online gaming':ab,ti OR 'internet gaming':ab,ti OR 'computer gaming':ab,ti OR 'video gaming':ab,ti OR 'gaming':ab,ti OR 'online-role playing gaming':ab,ti OR 'gaming behavior':ab,ti OR 'digital gaming':ab,ti) AND (abuse:ab,ti OR addiction:ab,ti OR compulsive:ab,ti OR dependence:ab,ti OR disorder:ab,ti OR effects:ab,ti OR excessive:ab,ti OR habit:ab,ti OR misuse:ab,ti OR overuse:ab,ti OR problem:ab,ti) AND [humans]/lim AND [english]/lim AND [embase]/lim AND [2020-2022]/py  Filters used: English, 2020-2022 |
| Scopus | ( TITLE-ABS-KEY ( "online gam*" OR "internet gam*" OR "computer gam*" OR "video gam*" OR "gaming" OR "online role-playing game" OR "gaming behavior" OR "digital gam*" ) AND TITLE-ABS-KEY ( abuse OR addiciton OR compulsive OR dependenc* OR disorder* OR effect* OR excessive OR habit* OR misuse OR pathological OR problem* OR problematic OR overuse ) AND TITLE-ABS-KEY ( "COVID-19" OR "COVID 19" OR "COVID 19 pandemic" ) ) AND ( LIMIT-TO ( PUBYEAR , 2022 ) OR LIMIT-TO ( PUBYEAR , 2021 ) OR LIMIT-TO ( PUBYEAR , 2020 ) ) AND ( LIMIT-TO ( LANGUAGE , "English" ) ) AND ( LIMIT-TO ( EXACTKEYWORD , "Human" ) OR LIMIT-TO ( EXACTKEYWORD , "Humans" ) ) Filters used: Human, English, 2020-2022 |
| CINAHL | ( TI "online gam*" OR AB "online gam*" OR TI "internet gam*" OR AB "internet gam*" OR TI "computer gam*" OR AB "computer gam*" OR TI "video gam*" OR AB "video gam*" OR TI "online-role playing game" OR AB "online-role playing game" OR TI "gaming behavio?r" OR AB "gaming behavio?r" OR TI "digital gam*" OR AB "digital gam*" ) AND ( TI abuse OR AB abuse OR TI addiction OR AB addiction OR TI compulsive OR AB compulsive OR TI dependenc* OR AB dependenc* OR TI disorder* OR AB disorder* OR TI effect* OR AB effect* OR TI excessive OR AB excessive OR TI habit* OR AB habit* OR TI misuse* OR AB misuse* OR TI pathological OR AB pathological OR TI problem* OR AB problem* OR TI overuse OR AB overuse ) AND ( (MH "COVID-19") OR TI "COVID-19" OR AB "COVID-19" OR TI "COVID 19" OR AB "COVID 19" OR TI "COVID 19 pandemic" OR AB "COVID 19 pandemic" )  Filters used: English and Pub Date: 2020-2022 |
| PsycNet | ((title: ("online gam*")) OR (abstract: ("online gam*")) OR (title: ("internet gam*")) OR (abstract: ("internet gam*")) OR (title: ("computer gam*")) OR (abstract: ("computer gam*")) OR (title: ("video gam*")) OR (abstract: ("video gam*")) OR (title: ("online role-playing game")) OR (abstract: ("online role-playing game")) OR (title: ("gaming behaviour")) OR (abstract: ("gaming behaviour")) OR (title: ("digital gam*")) AND (abstract: ("digital gam*"))) AND (((title: (abuse)) OR (abstract: (abuse)) OR (title: (addiciton)) OR (abstract: (addiciton)) OR (title: (compulsive)) OR (abstract: (compulsive)) OR (title: (dependenc*)) OR (abstract: (dependenc*)) OR (title: (disorder*)) OR (abstract: (disorder*)) OR (title: (effect*)) OR (abstract: (effect*)) OR (title: (excessive)) OR (abstract: (excessive)) OR (title: (habit*)) OR (abstract: (habit*)) OR (title: (misuse)) OR (abstract: (misuse)) OR (title: (pathological)) OR (abstract: (pathological)) OR (title: (problem*)) OR (abstract: (problem*)) OR (title: (problematic)) OR (abstract: (problematic)) OR (title: (overuse)) OR (abstract: (overuse))) AND (HasFullText: true)) AND ((title: ("COVID-19")) OR (abstract: ("COVID-19")) OR (title: ("COVID 19")) OR (abstract: ("COVID 19")) OR (title: ("COVID 19 pandemic")) OR (abstract: ("COVID 19 pandemic")))  Filters used: English and Pub Date: 2020-2022 |
