# Supplementary material for: Effect of COVID-19 pandemic on internet gaming disorder among general population: A systematic review and meta-analysis
Source: PLOS Glob Public Health. 2023 Apr 7;3(4):e0001783. doi: 10.1371/journal.pgph.0001783 (PMC10081738; doi:10.1371/journal.pgph.0001783)
Supplement: Attachment — Submitted filename: Response to Reviewers (1).docx [file pgph.0001783.s003.docx]

Dear Editor and Reviewers,

Thank you for your positive responses. We have tried our best to address all your comments and revised the manuscript as suggested. Below please find the point-by-point responses to each comment. We look forward to your positive response.

**Response to the Editor and reviewers**

|  | **Editor Comments** | **Response to comments** |
| --- | --- | --- |
| 1. | Some of the studies appear to be from the same author group (e.g., references 22, 29 and 31). Were authors able to assess whether these papers were reporting outcomes for different populations? If the populations are the same (i.e., same study sample), how did the authors arrive at a decision to include papers in the final analysis? | Thank you for your input on this statement. The studies were conducted in different time periods with different sample sizes. However, there has been overlap of populations in different studies. We have removed the studies with overlapping population from the meta-analysis. We have selected only one study out of three studies from the same author groups that clearly compared the timelines before the pandemic in 2019 and during the pandemic. |
| 2. | Authors may also want to strengthen their discussion of the implications of the study in lines 343-351. In addition to mentioning what the findings mean for practitioners working with IGD-affected individuals and policymakers who might regulate internet gaming in a pandemic context, it might also be important to mention how readers should situate the findings in the now-normal period. | Thank you for your comment. We have tried our best to strengthen the implications of our study.  (Discussion Line 357-372 of the revised manuscript)  We have also revised the last paragraph of Introduction (Line 76-87). |
|  | **Reviewers' comments:** |  |
|  | **Reviewer #1**: |  |
|  | **Abstract** |  |
| 1. | “home confinement during COVID-19 may have contributed to this to some extent”, please rephrase this sentence in accordance with your findings, as the matter is past now and you might have the recorded effects in this regard. | Thank you for your input on this statement. We have changed the sentence in the manuscript.  (Abstract, page 2, Line 26-31) |
| 2. | “Our study aims to assess”, please use past tense everywhere. | Thank you for your input in this statement. We have rephrased this into past tense as you suggested. (Abstract, line 30) |
| 3. | The reader does not get a clear-cut concluding statement out of the abstract, what came out of this study? Did the pandemic effect IGD and how much? | We have revised the abstract and tried to make it clearer. (Abstract, page 3, Line 44-47)  “Our study showed no clear evidence of increased IGD during COVID-19; however, the pooled prevalence was 8 % during the pandemic. The limited number of comparable studies, substantial heterogeneity, and low certainty of evidence made it difficult to draw definitive conclusions.” |
|  | **Introduction:** |  |
| 1. | Why were the researchers interested only in the IGD? Please provide a solid rationale for this study. | Thank you for your comment. We have included a rationale for the study as suggested.  (Introduction, Line 76-87) |
| 2. | The last paragraph of Introduction seems as if it is written in a hurry. Please decorate it further with a justified significance and scope of this study. | Thank you for your comment.  We have revised the last paragraph of the introduction to highlight the significance and scope of the study.  (Introduction, Line 76-87) |
|  | **Results** |  |
| 1. | Line spacing within the tables can be reduced to have a better symmetry. | Thank you for the suggestion. We have reduced the line spacing in the tables. |
| 2. | Table 3 seems to be the main finding of the study. However, it does not signify the statistically proven effect of the pandemic on IGD. How the authors would determine the “effect” without a relevant statistical procedure such as regression, t-test, or ANOVA. | Thank you for pointing this. We agree that the values in Table 3 does not determine “the effect” as there is no relevant statistical procedure such as regression, t-test, or ANOVA. We have revised the term “effect” to “outcome measures” as the values reflect mean difference, pooled mean, and pooled prevalence. |
|  | **Conclusion:** |  |
| 1. | “Therefore the evidence on the prevalence of IGD during COVID-19 remains inconclusive”, having said that the authors must focus more on the need and rationale of the study, what made them think that IGD might have been increased during the pandemic and why the results came otherwise? | We have added the rationale of the study explaining about the research gap and how it has led to the lack of evidence.  (Conclusion, Line 373-384) |
| 2. | “Nevertheless, this systematic review and meta-analyses highlight the research gap for emerging mental health problems such as IGD during the pandemic”, please reconsider this statement as thousands of mental health related studies have already been carried out during the pandemic. | Thank you for your comment. We have revised this sentence and focused specifically on IGD which is a recently recognized mental health condition.  (Conclusion, Line 379-381) |
| 3. | Authors should write some feasible suggestions based on their findings. For instance, they can focus more on the addictive properties of IGD that are quite effective even in the non-pandemic normal situations and have the capacity to disturb the daily routines. | Thank you for your comment. We have explained the addictive properties of IGD and also how it may continue beyond pandemic. We have added these statements in Introduction (Line 76-87) and Discussion (Line 368-366). |
|  | **Reviewer #2:** |  |
|  | I commend the authors for writing such an interesting article.  The manuscript paper does have a few flaws, and the text might benefit from more thorough editing. I've reviewed it carefully and have some improvements to suggest. Please address the following comments and recommendations to strengthen your manuscript. | Thank you for your positive comment and your valuable suggestions.  We have tried our best to address all the comments and recommendations and strengthen the manuscript. |
| 1. | Please clarify if the data extraction sheet was pilot tested before actual use. If yes, please note this in your manuscript. Also, provide more details about literature summary tables and how they were developed and tailored to meet the review needs | Thank you for your input. The table was adapted from the format of the data extraction table recommended by Cochrane. We have added this to data extractions section. (Methods, Line 118) |
| 2. | Your rationale/ justification section needs to address the research gap in the intended field so that it reflects why this study is important to conduct now. | Thank you for your comment we have tried our best to strengthen the rationale in the introduction last paragraph.  (Introduction, Line 76-87) |
| 3. | The novelty of your work must be discussed, it seems it is weak | We have tried our best to improve it. |
| 4. | In the last paragraph of the introduction, you should fully explain why you used systematic review and meta-analysis | Thank you for your comment we have included why we used systematic review and meta-analysis in the introduction.  (Introduction, Page 6, 115-116) |
| 5. | There are some spelling and grammatical errors in the text. Please correct them | We have reviewed the manuscript and corrected the spelling and grammatical errors. |
| 6. | In the result and discussion section, more interpretations are needed -Emphasize the new research findings and mention what your research added to the previous research | Thank you for your valuable comment. We have revised the result and discussion section accordingly. |
